# Patient-reported outcomes of esthetics, function and oral hygiene with single dental implants 10–15 years after placement: a cross-sectional study

**DOI:** 10.2340/aos.v84.42724

**Published:** 2025-01-21

**Authors:** Viveca Wallin Bengtsson, Christel Lindahl, Sven Scholander

**Affiliations:** a Department of Oral Health, Faculty of Oral Health Science, Kristianstad University, Kristianstad, Sweden; b Private Practice, Kristianstad, Sweden

**Keywords:** Dental esthetics, patient-reported outcome measurements, peri-implant mucositis, peri-implantitis, single dental implant

## Abstract

**Aims:**

Little attention has been paid to patients’ perception of function and esthetics with single dental implants. The aim of this study was therefore to describe patient-reported function and esthetic outcomes in single dental implants. A second aim was to study the objective esthetics in single dental implants.

**Material and methods:**

Patients with one single dental implant in the esthetic zone were selected. Two questionnaires with visual analog scales (VAS) were filled in by the patients, and intraoral photographs were taken. One of the questionnaires related to satisfaction with cleaning and function, and the other involved the esthetics of the single dental implant. One dentist reviewed the photographs using the pink esthetic score/white esthetic score (PES/WES) index.

**Results:**

For chewing and for speaking, the scores were 8.8 and 9.9 respectively on a VAS (best 10). The overall esthetic score on a VAS was 8.6 (best 10). The PES/WES in the present study was 14.6 (standard deviation [SD] ± 1.9), and 3/45 (6.7%) of the single dental implants never reached clinical acceptability.

**Conclusions:**

Patients reported high satisfaction with both the function and the esthetics of single dental implants. Both subjectively and objectively, the peri-implant mucosa was less favorable compared with the crown.

## Introduction

Dental implant treatment is a commonly used therapy for replacing missing teeth [1]. Single dental implants have become the most common type of implant therapy [2]. Although many studies have focused on the clinical outcomes of single dental implant treatments [3–6], there is a need for future research to more thoroughly address patients’ perceptions of these implants. The VIII European Workshop on Periodontology highlights the importance of patient-reported outcome measurements (PROMs), peri-implant tissue health and functional and esthetic outcomes related to implant-supported reconstructions [7].

Patients’ expectations of the esthetic outcome of dental implant treatments are particularly high when implants are placed in the esthetic zone [8]. A meta-analysis in a systematic review showed a 5-year cumulative soft tissue complication rate of 7.1% for implant-supported single crowns with unacceptable esthetics [1]. A major clinical challenge is creating a harmonious mucosal margin without abrupt changes in tissue height and achieving a convex contour of the alveolar crest [9]. The absence of inter-tooth-implant papilla results in a ‘black triangle’ space which negatively impacts the esthetic appearance [10].

Esthetic evaluations can be conducted both objectively and subjectively. Subjective evaluations which focus on the patient’s perception of the esthetics can be made using questionnaires in which satisfaction or dissatisfaction can be expressed by the patient [11]. Few studies in early literature have examined patient satisfaction with single dental implants [12]. Evaluations of implant treatment should reflect patient opinions, as they represent one part of the effectiveness of care [13]. Conversely, objective evaluations of esthetics following implant therapy are performed by professional examiners. An objective evaluation is based on predefined criteria related to the harmonious appearance and the integration of the restoration with the rest of the patient’s dentition [14]. For a more comprehensive assessment of the esthetic outcome, both objective and subjective evaluations should be included [15].

Patients treated with a single dental implant often have high esthetic expectations [16–18]. However, few studies have evaluated functional and esthetic outcomes from a patient-centered perspective [14, 18–21]. Understanding patients’ perceptions of the function and esthetics of single dental implants would improve satisfaction and potentially enhance oral-health-related quality of life. The aim of the study was therefore to describe the patient-reported function and esthetic outcomes in single dental implants 10–15 years after placement. A second aim was to study objective esthetics in single dental implants 10–15 years after placement.

## Material and methods

### Study design

The present cross-sectional study is based on a clinical investigation of the esthetic and biological status of single dental implants, as well as patient-reported outcome measures (PROMs), at a 10- to 15-year follow-up.

Periodontists and oral surgeons performed the surgical procedures in two stages. The implants were submerged with the uppermost part of the fixture with the marginal bone, and healing screws were positioned. A titanium healing abutment was inserted 6–9 months after initial surgery. Four to five weeks later, the healing abutment was replaced by a permanent single cementable dental abutment of appropriate length. The permanent abutment was tightened with a gold screw at 32 Ncm using a torque driver (Nobel Biocare AB), and all the crowns were cemented. The cementable abutments used were either standardized titanium abutments or single direct-cast abutments (085-089), and the crowns were of either all-ceramic (Cera-One) or metal-ceramic (MK) design.

The study was approved by the Regional Ethics Board in Lund (Dnr 300/2006) and conducted in accordance with the World Medical Association’s Declaration of Helsinki. Additionally, the study adhered to the ‘Strengthening the Reporting of Observational Studies in Epidemiology’ (STROBE) guidelines.

### Patient selection

Patients undergoing prosthetic single dental implant treatment at an Oral Prosthodontic Specialist Clinic in Kristianstad (Folktandvården, Public Dental Service, Kristianstad, Sweden) between 1991 and 1996 were consecutively invited to participate in the study. These patients were examined by an experienced dental hygienist in 2006–2007. Inclusion criteria specified that participants must have only one single dental implant reconstruction in the esthetic zone, defined as the region from the upper right canine to the upper left canine. Study participants were also required to have completed two questionnaires, participated in an oral interview, and had intraoral photographs taken. Patients were excluded if they had an edentulous area adjacent to the implant being studied. All participants were informed of the study procedures and they provided written informed consent before participating in the study.

### Collection of data from an oral questionnaire

An updated medical history focusing on smoking habits and reasons for tooth loss (including aplasia, trauma, fracture, periodontitis, and endodontic complications) was obtained. Additionally, the patient answered questions regarding supportive care, specifically the frequency of visits each year, and whether these treatments were performed by a dentist or a dental hygienist.

### Patient reported outcomes

Patients were asked to complete two different questionnaires related to their satisfaction with the treatment received. They indicated their satisfaction using a visual analog scale (VAS), which consisted of a straight horizontal line graded from 0 to 10. The mean visual analog score was then calculated for each patient based on their responses.

#### Questionnaire 1

This questionnaire focused on the function and cleaning ability of the single dental implant, with various statements adopted from Pjetursson et al. [22]. The VAS ranged from 0 on the left end, indicating ‘no, absolutely not’, to 10 on the right end, indicating ‘yes, absolutely’. The statements were as follows:

1. My single implant functions very well and I can chew on it very well.
2. I feel more secure biting on my teeth compared with my implant-supported crown.
3. To speak, I can easily use my implant-supported crown/bridge.
4. I can clean my single implant very well.
5. It is easier for me to clean my single implant than to clean my teeth.
6. I need more time to clean my implant than to clean my teeth.
7. The tissues around my single implant bleed less than those around my teeth.
8. I got exactly what I expected.
9. I would like this treatment again, if needed.
10. I would recommend this treatment to a friend or relative, if indicated.

#### Questionnaire 2

The second questionnaire evaluated the patient’s perception of the esthetics of the single dental implant. On the VAS, the left end indicated 0, representing ‘worst possible’, while the right end indicated 10, representing ‘best possible’. The mean VAS score was calculated for each patient based on their responses in the following areas:

1. Crown color
2. Crown shape
3. Mucosal appearance
4. Overall satisfaction

### The Pink Esthetic Score/White Esthetic Score index

Each implant was photographed using a digital camera, capturing both a frontal view and a close-up of the crown. The contralateral reference tooth was included in the images to ensure compatibility. A dentist (VWB) reviewed the intraoral photographs using the Pink Esthetic Score (PES) and White Esthetic Score (WES) index [16]. The PES/WES assesses various parameters, including mesial papilla fill, distal papilla fill, curvature of the facial mucosa, level of the facial mucosa, root convexity, and the color and texture of the soft tissue, as well as crown form, volume/outline, color, surface texture, and translucency. Each parameter is scored from 0 to 2, with a score of 2 representing the best outcome and 0 the worst. Papilla scores are evaluated based on the completeness (score 2), incompleteness (score 1), or absence of papillary tissue (score 0). The curvature of the facial soft-tissue line is assessed as identical (score 2), slightly different (score 1), or markedly different (score 0) compared to a reference tooth, typically the contralateral tooth. The level of the facial mucosa is scored as identical vertical level (score 2), with a slight discrepancy (≤1 mm) receiving a score of 1, and a major discrepancy (≥1 mm) receiving a score of 0.

**Figure 1:**
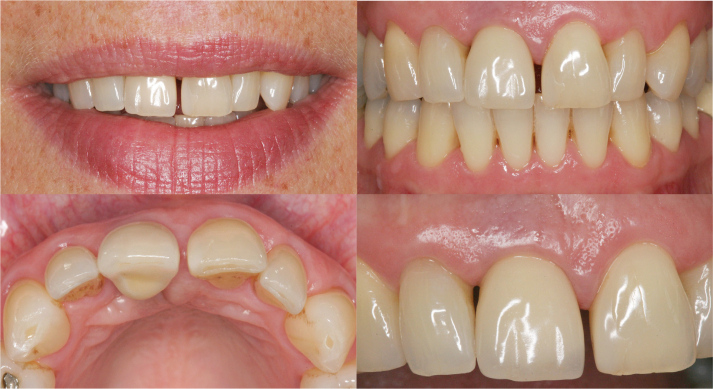
A patient with a single dental implant in the region of tooth 11, 13 years after placement. Pink esthetic score (PES) = 5, White esthetic score (WES) = 8, according to Belser et al. 2009. The patient’s perception of the esthetics on a visual analogue scale (VAS) = 40.

Additionally, the presence (score 2), partial presence (score 1), or absence (score 0) of a convex profile, as well as the color and texture of the facial mucosa, are assessed based on the criteria outlined by Belser et al. [16]. A score of 2 for this combined variable requires all three conditions to match the reference tooth. Under optimal conditions, the maximum PES score is 10, while a PES score of 6 is deemed clinically acceptable. For the WES, the maximum score is also 10, with a score of 6 considered clinically acceptable (Figure 1) [17].

The intraclass correlation coefficient (ICC) for the PES/WES index between two observer measurements was 0.89 (range: 0.58–0.97, *p* = 0.00) based on a total of 10 observations.

### Clinical examination

Probing pocket depth (PPD) was measured at the implant sites using a Hawe Click-Probe (Kerr-Hawe SA, Bioggio, Switzerland), with measurements taken at four sites per implant. PPD values were then recorded. Additionally, bleeding on probing (BOP) was registered at each of the four sites per implant and tooth 30 s following PPD measurement and was expressed as a percentage of the evaluated sites. Finally, the presence of suppuration was assessed at the four sites per implant.

Peri-implant mucositis was diagnosed based on the presence of BOP and/or suppuration following gentle probing, along with an absence of bone loss beyond the crestal bone level changes associated with initial bone remodeling at the implant site. In this study, this was defined as ≤2.5 mm apical of the most coronal portion of the intra-osseous part of the implant. This definition aligns with the current guidelines on Peri-Implant Diseases and Conditions [23].

Peri-implantitis was diagnosed based on the presence of bleeding on gentle probing (BOP) and bone levels measuring ≥3 mm apical of the most coronal portion of the intra-osseous part of the implant. This aligns with the latest standards for classifying Peri-Implant Diseases and Conditions [23].

### Radiographic examination

Analog periapical and bitewing radiographs were taken of the implants using the parallel technique. These images were then digitized and analyzed with *synedra View Personal* dental imaging software to assess alveolar bone loss. Measurements were taken from the implant shoulder to the highest point of the marginal bone level on both mesial and distal surfaces. To ensure measurement consistency, intra-observer reliability was evaluated by having the same observer (VWB) repeat measurements on 15 randomly selected implants after 4 months. The ICC was found to be 0.97 for the mesial implant surfaces (95% confidence interval [CI]: 0.92–0.99, *p* < 0.000) and 0.98 for the distal surfaces (95% CI: 0.95–0.99, *p* < 0.000).

### Statistical analysis

For statistical analysis, data were processed using the *Statistical Package for the Social Sciences (SPSS) Predictive Analytics Software* (PASW) version 28.0 (SPSS Inc., Armonk, NY, USA) on a personal computer. Descriptive statistics, including mean values and standard deviations (SD), were calculated. Pearson’s χ² test and Mantel-Haenszel common odds ratios were applied for dichotomous data analysis, while categorical data were expressed in percentages. Estimates such as odds ratios and mean differences were provided alongside corresponding 95% confidence intervals. The *p*-value significance threshold was set at 0.05. No data was missing. Cronbach’s alpha was applied to assess the reliability of the PES/WES index for the objective evaluation of esthetic outcomes.

## Results

At the 10–15-year follow-up examination conducted in 2005–2006, 58 patients were evaluated by an experienced dental hygienist. Of these, 45 patients with a single dental implant met the inclusion criteria. One patient who had lost an implant 1.5 years after the prosthetic reconstruction was completed was included in the study because the implant was replaced and followed up 13 years later. Among the 45 patients, 19/45 (42.2%) were women. The mean age of the patients was 40 years (±10.5, range 30–80 years). There were 9/45 (20.0%) current smokers among the patients. All the implants carried the Brånemark system (Nobel Biocare AB), and the cementable abutments were standardized titanium abutments in 41/45 (91.1%) and single abutment direct cast abutment (DCA) 085-089 in 4/45 (8.9%). The single crowns were all ceramic in 38/45 (84.4%), and the remaining 7/45 (15.6%) were metallic ceramic. The single dental implant had been in use for 12.6 years (±1.39, range 10–15).

Trauma (73.3%), aplasia (24.4%), and decayed teeth (2.3%) were the reasons for tooth loss and the indication for implant treatment.

### Patient reported outcome measures

#### Function and cleaning ability of the single dental implant

Highest satisfaction was reported for the chewing function, with a mean score of 8.8 (±2.5, range 0–10) and a median of 10.0, followed by speech function, with a mean rating of 9.9 (±0.5, range 7–10) and a median of 10.0, and cleaning ability, with a mean score of 9.2 (±1.9, range 2–10) and a median of 10.0. The mean, median, and range for each statement are provided in Table 1.

**Table 1:** Satisfaction with function and cleaning ability of the single dental implant on a Visual Analog Scale.

Statement	Mean SD Range	Median
1. My single implant functions very well and I can chew on it very well.	8.8 ± 2.5 (range 0–10)	10.0
2. I feel more secure biting on my teeth compared with my implant-supported crown.	2.8 ± 3.5 (range 0–10)	0.0
3. To speak, I can easily use my implant-supported crown/bridge.	9.9 ± 0.5 (range 7–10)	10.0
4. I can clean my single implant very well.	9.2 ± 1.9 (range 2–10)	10.0
5. It is easier for me to clean my single implant than to clean my teeth.	2.5 ± 3.0 (range 0–10)	0.0
6. I need more time to clean my implant than to clean my teeth.	1.3 ± 2.6 (range 0–10)	0.0
7. The tissues around my single implant bleed less than those around my teeth.	4.4 ± 3.8 (range 0–10)	5.0
8. I got exactly what I expected.	8.2 ± 2.3 (range 3–10)	9.0
9. I would like this treatment again, if needed.	9.4 ± 1.5 (range 3–10)	10.0
10. I would recommend this treatment to a friend or relative, if indicated.	9.6 ± 1.2 (range 3–10)	10.0

SD: standard deviation.

Patient-reported statements 0 = no, absolutely not; 10 = yes, absolutely.

#### Esthetics of the single dental implant

Patients were most satisfied with the color and shape of the single crown. Less satisfaction was felt with the mucosal margin surrounding the implant. Overall satisfaction with the esthetics of the implant was a mean of 8.6 (±1.3, range 6–10) and the median value was 9.0 (Table 2).

**Table 2:** Patient-perceived esthetics of the single dental implant according to a Visual Analog Scale (mean, SD, range and median).

	All patients (*n* = 45)	Patients with peri-implant mucositis (*n* = 40)	Patients with peri-implantitis (*n* = 2)
Crown color	9.2 ± 1.1 (6–10) 10.00	9.3 ± 1.1 (7–10) 10.0	7.5 ± 2.1 (6–9) 7.5
Crown shape	9.2 ± 1.4 (5–10) 10.00	9.3 ± 1.4 (5–10) 10.0	8.5 ± 2.1 (7–10) 8.5
Mucosal appearance	7.1 ± 2.8 (2–10) 8.00	7.2 ± 2.7 (2–10) 8.0	5.5 ± 3.5 (3–8) 5.5
Overall satisfaction	8.6 ± 1.3 (6–10) 9.00	8.7 ± 1.2 (6–10) 9.0	7.5 ± 2.1 (6–9) 7.5

SD: standard deviation; *n*: number.

Patient-perceived esthetics: 0 = not satisfied at all; 10 = completely satisfied.

### The PES and the WES index

The overall PES had a mean score of 6.2 (±1.4, range 3–9) and the median 6.0. Of the patients, 13/45 (29%) had a PES score of <6. The corresponding figure for the WES was a mean of 8.4 (±1.2, range 5–10) and a median of 9.0 (Table 3). A WES score of <6 was reported in 1/45 (2.2%).

**Table 3:** Mean pink and white esthetic scores for the single dental implants.

PES	Mesial papilla	Distal papilla	Curvature of facial mucosa	Level of facial mucosa	Root convexity, soft tissue color and texture	Total PES (max 10)
Mean	1.2	1.0	1.6	1.3	1.1	6.2
SD	0.6	0.5	0.7	0.8	0.4	1.4
Median	1.0	1.0	2.0	1.0	1.0	6.0

WES	Tooth form	Tooth volume	Color (hue/value)	Surface texture	Translucency and characterization	Total WES (max 10)

Mean	1.7	1.2	1.6	2.0	1.9	8.4
SD	0.5	0.7	0.5	0.2	0.3	1.2
Median	2.0	1.0	2.0	2.0	2.0	9.0

PES: pink esthetic score; WES: white esthetic score; SD: standard deviation.

0 = worst outcome and 2 = best outcome reported by a dentist (VWB)

The combined PES/WES had a mean of 14.6 (±1.9, range 9–18) and a median of 15.0. A score of <12 for the PES/WES was noted in 3/45 (6.7%) patients.

### Peri-implant status

A diagnosis of peri-implant mucositis was made in 40/45 (88.9%) of the implants and a diagnosis of peri-implantitis in 2/45 (4.4%). The mean PPD of the implants was 3.8 mm (SD 0.9) and 22.2% of PPD were ≥6 mm. Annual visits to the dental hygienist for supportive treatment were reported by 1 out of 45 patients (2.2%), and the corresponding figure for visits to the dentist was 30 out of 45 patients (66.7%).

### Peri-implant diseases and esthetics of the single dental implant

Patients with peri-implant mucositis reported an overall esthetic satisfaction of 8.7 ± 1.2 (range 6–10), and the median value was 9.0. For patients with peri-implantitis, the overall esthetic satisfaction was 7.5 ± 2.1 in mean (range 6–9) and the median value 7.5 (Table 2).

### Peri-implant diseases and the PES and the WES index

In patients with peri-implant mucositis, the PES score was 6.1 ± 1.4 (range 3–9) and the median was 6.0; the WES score was 8.5 ± 1.0 (range 6–10) and the median was 9.0. The combined PES/WES score was 14.7 ± 1.8 (11–18) and the median was 15.0 (Table 2).

For patients with peri-implantitis, the PES was 7.0 ± 1.4 (range 6–8) and the median was 7.0, and the WES score was 7.5 ± 2.1 (range 6–9) with the median 7.5. The combined PES/WES score was 14.5 ± 0.7 (14–15) and the median was 14.5.

## Discussion

In this study, subjective evaluations of single dental implants function showed a score of 8.8 for chewing and 9.9 for speaking, on a VAS with 10 indicating absolute agreement. For esthetic satisfaction, overall esthetics received a score of 8.6 on the VAS with crown color and crown shape achieving the highest ratings (9.2 for each), with 10 indicating absolute agreement. The surrounding mucosa obtained the lowest score, that is, 7.1 on the VAS.

In a study by Wang et al. [19], patients with implant crowns and PROMs 10 years after implant placement reported that 91.6% were satisfied with their chewing ability, and the remaining 8.4% of patients reported slight restrictions. These results align with the high levels of chewing satisfaction observed in the present study. In our study, the patients reported that their speaking ability after implant therapy was close to the level of absolute agreement. This is consistent with Pjetursson et al.’s study [22], which found that 2% of the patients reported phonetic problems 5–15 years after implant installation, an incidence similar to our study.

The perceived esthetics of maxillary anterior single implants in this study aligns with previous findings by Altay et al. [24], though the questions to the patients were not entirely identical. A tendency for peri-implantitis to reduce esthetic satisfaction was noted, but with only 2 out of 45 patients diagnosed with peri-implantitis; this should be interpreted cautiously.

The mean combined PES and WES in the present study was 14.6 (SD ± 1.9). In a study by Belser et al. [16], the same index was used to evaluate maxillary anterior single dental implants in 45 patients but only over a 2 to 4-year follow-up period. Strikingly, the PES/WES score was similar, with a score of 14.7 (SD ± 1.18). In the present study, 3 out of 45 (6.7%) patients never reached clinical acceptability according to the threshold of 12 as established by Belser et al. [16]. The PES score had a mean of 6.2, and 13 out of 45 (29%) patients were below the threshold for clinical acceptability of <6, whereas the WES score had a mean of 8.4 and only 1 out of 45 (2.2%) of the patients was below the threshold for clinical acceptability, that is, <6. These results indicate that the esthetics of the surrounding mucosa were rated lower than the esthetics of the implant-supported crown. In a study by Cho et al. [25] similarly evaluated 41 patients with single dental implants were using the PES/WES by eight observers. The mean PES had 5.17 scores, whereas the WES had 6.02. Although the values in Cho et al.’s [25] study were generally lower, both studies showed a trend of lower PES compared to WES. In Cho et al.’s study, periodontitis was the main reason for natural tooth extractions with subsequent loss of bone and interproximal crest height should determine the presence or absence of the peri-implant papilla [25]. In the present study, the approximal papilla scores were 1.2 for the mesial papilla and 1.0 for the distal papilla. Previous studies have shown that papilla height largely depends on the bone level height at adjacent root surfaces [26, 27], which was not investigated in the present study. The papilla scores in the present study were between those of Cho et al. [25] (0.63 mesial and 0.64 distal) and Belser et al. [17] (1.6 mesial and 1.3 distal). Unlike Belser et al. [16] who employed an early placement protocol, both our study and Cho’s used a late placement protocol, which may influence papilla fill, as early implant placement tends to yield better initial papilla fill but achieves similar interproximal fill over time [27].

The reason there are some differences between the papilla variable could depend on the follow-up period, which was the longest in our study. Our study’s follow-up period was 10 years or more, compared to that of Cho et al. [25] (1–8 years) and Belser et al. [16] (2–4 years). Additionally, the implant locations varied; in the present study, implants were positioned in central and lateral incisors as well as canines, whereas Belser et al. [16] and Cho et al. [25] included first premolar positions.

One strength of this study is the use of the combined PES/WES index to objectively assess both the surrounding mucosa and the implant-supported crown, as it is known for its high reproducibility [15]. The PES/WES was reassessed with a fair ICC, which supports its consistency and reliability. Notably, the PES was identified as one of the two most validated esthetic indices for single dental implants by the 6th EAO Consensus Conference in 2021 [28]. However, a limitation is that only one dentist conducted the measurements. Including multiple evaluators could have made the findings more representative.

A limitation of this study is that the PROMs used for assessing cleaning and functional ability, as well as esthetics, were based on a non-standardized and non-validated questionnaire and VAS. This lack of standardization and validation is a noted weakness. Currently, to our knowledge, there is no validated, implant-specific patient-reported outcome measure focusing on esthetic evaluation, which understandably affects the reliability of the findings [14, 18]. The subjective tool used by patients, VAS, highlights the individualized nature of esthetic perception, which can be shaped by factors such as age, gender, educational background, and psychological well-being, and may not always align with an objectively ideal esthetic outcome [15]. Additionally, the PROMs in this study reflect materials and techniques from the 1990s, which may not be directly comparable to those used today, thereby limiting the generalizability of these findings.

At the 10-year follow-up, patients with single dental implants reported satisfaction with both chewing and speaking functions. They also expressed overall high satisfaction with esthetics, including the peri-implant mucosa and the implant crown. From an objective standpoint, the esthetics of most single dental implants and surrounding peri-implant mucosa were deemed clinically acceptable. The PES, however, was slightly less favorable than the WES, suggesting that the esthetic outcome of the mucosa could be improved, indicating a need for more treatment focus on the peri-implant mucosa. Combining both subjective and objective assessments of single dental implants is essential for a comprehensive esthetic evaluation from both patient and clinician perspectives.

## Conclusions

In this small study, patients reported high satisfaction with the cleaning, chewing ability, and overall esthetics of their single dental implants. However, satisfaction with the surrounding peri-implant mucosa was less favorable than with the implant crown, as reflected in both subjective and objective assessments.

## Data Availability

The data supporting the findings in this study are available in response to a reasonable request.

## Protocol registration statement

A study protocol was prepared before the study and was followed throughout the study. A registration of the protocol was never performed.

## References

[1] Jung RE, Zembic A, Pjetursson BE, Zwahlen M, Thoma DS. Systematic review of the survival rate and the incidence of biological, technical, and aesthetic complications of single crowns on implants reported in longitudinal studies with a mean follow-up of 5 years. Clin Oral Implants Res. 2012;23(Suppl 6):2–21. 10.1111/j.1600-0501.2012.02547.x 23062124

[2] Brugger OE, Bornstein ME, Kuchler U, Janner S, Chappuis V, Buser D. Implant therapy in a surgical specialty clinic: an analysis of patients, indications, surgical procedures, risk factors, and early failures. Int J Oral Maxillofac Implants. 2015;30:151–60. 10.11607/jomi.3769 25506641

[3] Bormann KH, Gellrich, NC, Kniha, H, Schild S, Weingart. D, Gahlert, M. A prospective clinical study to evaluate the performance of zirconium dioxide dental implants in single-tooth edentulous area: 3-year follow-up. BMC Oral Health. 2018;18:181. 10.1186/s12903-018-0636-x 30382850 PMC6211599

[4] Derks, J, Schaller, D, Håkansson, J, Wennström, JL, Tomasi, C, Berglundh, T. Effectiveness of implant therapy analyzed in a Swedish population: prevalence of peri-implantitis. J Dent Res. 2016;95:43–9. 10.1177/0022034515608832 26701919

[5] Donati M, Ekestubbe, A, Lindhe, J, Wennström, JL. Implant-supported single-tooth restorations. A 12-year prospective study. Clin Oral Implants Res. 2016;27:1207–11. 10.1111/clr.12726 26577573

[6] Scarano A, Conte, E, Mastrangelo F, Greco A, Lucchina A, Lorusso, F. Narrow single tooth implants for congenitally missing maxillary lateral incisors: a 5-year follow-up. J Biol Regul Homeost Agents. 2019;6(Suppl 2):69–76. 32425026

[7] Tonetti M, Palmer R. Working Group 2 of the VIII European Workshop on Periodontology Clinical research in implant dentistry: study design, reporting and outcome measurements: consensus report of Working Group 2 of the VIII European Workshop on Periodontology. J Clin Periodontol. 2012;39(Suppl 12):73–80. 10.1111/j.1600-051X.2011.01843.x 22533948

[8] Pradyachaipimol N, Tangsathian T, Supanimitkul K, Sophon N, Suwanwichit T, Manopattanasoontorn S, et al. Patient satisfaction following dental implant treatment: a survey. Clin Implant Dent Relat Res. 2023:25(3):613–23. 10.1111/cid.13196 36881004

[9] Belser UC, Bernard JP, Buser D. Implant-supported restorations in the anterior region: prosthetic considerations. Pract Periodontics Aesthet Dent 1996;8:875–83; quiz 884. 9242147

[10] Cardaropoli G, Lekholm U, Wennström JL. Tissue alterations at implant-supported single-tooth replacements: a 1-year prospective clinical study. Clin Oral Implants Res. 2006;17:165–71. 10.1111/j.1600-0501.2005.01210.x 16584412

[11] Gehrke P, Lobert M, Dhom GJ. Reproducibility of the pink esthetic score – rating soft tissue esthetics around single-implant restorations with regard to dental observer specialization. J Esthet Restor Dent. 2008;20:375–84; discussion 385. 10.1111/j.1708-8240.2008.00212.x 19120783

[12] den Hartog, Slater JJ, Vissink A, Meijer HJ, Raghoebar GMJ. Treatment outcome of immediate, early and conventional single-tooth implants in the aesthetic zone: a systematic review to survival, bone level, soft-tissue, aesthetics and patient satisfaction. J Clin Periodontol. 2008;35:1073–86. 10.1111/j.1600-051X.2008.01330.x 19040585

[13] Carr A, Wolfaardt J, Garrett N. Capturing patient benefits of treatment. Int J Oral Maxillofac Implants. 2011;26(Suppl):85–92; discussion 101–2. 21465001

[14] Lang NP, Zitzmann NU. Working group 3 of the VEWOP. Clinical research in implant dentistry: evaluation of implant-supported restorations, aesthetic and patient-reported outcomes. J Clin Periodontol. 2012;39(Suppl 12):133–8. 10.1111/j.1600-051X.2011.01842.x 22533953

[15] Stefanini M. Felice P, Mazzotti C, Mounssif I, Marzadori M, Zucchelli G. Esthetic evaluation and patient-centered outcomes in single-tooth implant rehabilitation in the esthetic area. Periodontol 2000. 2018;77:150–64. 10.1111/prd.12215 29493024

[16] Belser UC, Grütter L, Vailati F, Bornstein MM, Weber HP, Buser D. Outcome evaluation of early placed maxillary anterior single-tooth implants using objective esthetic criteria: a cross-sectional, retrospective study in 45 patients with a 2- to 4-year follow-up using pink and white esthetic scores. J Periodontol. 2009;80:140–51. 10.1902/jop.2009.080435 19228100

[17] Petsos H, Trimpou G, Eickholz P, Lauer HC, Weigl P. The influence of professional competence on the inter- and intra-individual esthetic evaluation of implant-supported crowns in the anterior maxilla. Clin Oral Implants Res. 2017;28:453–60. 10.1111/clr.12819 27009805

[18] McGrath C, Lam O, Lang N. An evidence-based review of patient-reported outcome measures in dental implant research among dentate subjects. J Clin Periodontol. 2012;39(Suppl 12):193–201. 10.1111/j.1600-051X.2011.01841.x 22533956

[19] Wang Y, Bäumer D, Ozga AK, Körner G, Bäumer A. Patient satisfaction and oral health-related quality of life 10 years after implant placement. BMC Oral Health. 2021;21:30. 10.1186/s12903-020-01381-3 33446161 PMC7807859

[20] Sermsiripoca K, Pisarnturakit PP, Mattheos N, Pimkhaokham A, Subbalekha K. Comparing pre- and post-treatment patients’ perceptions on dental implant therapy. Clin Implant Dent Related Res. 2021;23:769–78. 10.1111/cid.13036 34346146

[21] Gjelvold B, Chrcanovic BR, Bagewitz IC, Kisch J, Albrektsson T, Wennerberg A. Esthetic and patient-centered outcomes of single implants: a retrospective study. Int J Oral Maxillofac Implants. 2017: 32(5):1065–73. 10.11607/jomi.5495 28334057

[22] Pjetursson BE, Karoussis I, Bürgin W, Brägger, U, Lang NP. Patient´s satisfaction following implant therapy. A 10-year prospective cohort study. Clin Oral Implants Res. 2005;16:185–93. 10.1111/j.1600-0501.2004.01094.x 15777328

[23] Berglundh T, Armitage G, Araujo MG, Avila-Ortiz G, Blanco J, Camargo PM et al. Periodontal health and gingival diseases and conditions on an intact and a reduced periodontium: consensus report of workgroup 1 of the 2017 World Workshop on the Classification of Periodontal and Peri-Implant Diseases and Conditions. J Periodontol. 2018;89(Suppl 1):S74–84. 10.1002/JPER.17-0719 29926944

[24] Altay MA, Sindel A, Tezerişener HA, Yıldırımyan N, Özarslan MM. Esthetic evaluation of implant-supported single crowns: a comparison of objective and patient-reported outcomes. Int J Implant Dent. 2019;5:2. 10.1186/s40729-018-0153-3 30613918 PMC6321832

[25] Cho HL, Lee JK, Um HS, Chang BS. Esthetic evaluation of maxillary single-tooth implants in the esthetic zone. J Periodontal Implant Sci. 2010;40:188–93. 10.5051/jpis.2010.40.4.188 20827328 PMC2931307

[26] Choquet V, Hermans M, Adriaenssens P, Daelemans P, Tarnow DP, Malevez CJ. Clinical and radiographic evaluation of the papilla level adjacent to single-tooth dental implants. A retrospective study in the maxillary anterior region. Periodontol. 2001;72:1364–71. 10.1902/jop.2001.72.10.1364 11699478

[27] Schropp L, Isidor F, Kostopoulos L, Wenzel A. Interproximal papilla levels following early versus delayed placement of single-tooth implants: a controlled clinical trial. Int J Oral Maxillofac Implants. 2005;20:753–61. 16274150

[28] Thoma DS, Cosyn J, Fickl S, Jensen SJ, Jung RE, Raghoebar GM et al. Working group 2 of the 6th EAO Consensus Conference 2021. Soft tissue management at implants: summary and consensus statements of group 2. The 6th EAO Consensus Conference 2021. Clin Oral Implants Res. 2021;32(Suppl 21):174–80. 10.1111/clr.13798 34145925 PMC8596754

